# Genetic analysis of reciprocal differences in the inheritance of *in vitro* characters in pearl millet

**DOI:** 10.1590/1678-4685-GMB-2014-0380

**Published:** 2016

**Authors:** Valluri V Satyavathi, V. Manga, Muktinutalapati V. Subba Rao, Malladi Chittibabu

**Affiliations:** 1 Department of Botany, Andhra University, Visakhapatnam, India; 2 Department of Statistics, Andhra University, Visakhapatnam, India

**Keywords:** cytoplasmic inheritance, d_2_ dwarf, *in vitro* regeneration, pearl millet, reciprocal differences

## Abstract

Reciprocal differences persist in nature because of the unequal contribution of cytoplasmic determinants from male and female gametes to the zygote. The inheritance of genetic differences is an important factor that influences various traits, including somatic embryogenesis and regeneration *in vitro*. In this report, we estimate the cytoplasmic and maternal effects in pearl millet and their adequacy in describing the observed reciprocal differences based on an in depth study of the parents, F_2_s and reciprocal backcross progenies needed for fitting genetical models. Our study revealed that of the two characters examined, embryogenic callus quantity and regeneration frequency, the former showed a greater proportion of cytoplasmic nuclear interaction whereas the latter showed a greater role of nuclear factors. Additive-maternal effects influenced total callus quantity and dominance-maternal effects influenced total callus quantity, embryogenic callus quantity and regeneration frequency. Dwarfing was associated with the production of large quantities of embryogenic callus that had visually recognizable characteristics. The phenotypic nature of dwarf parents (green dwarf with long narrow leaves) with a genetic basis for a given character controlled by nuclear and cytoplasmic determinants can be exploited for other breeding programs.

## Introduction

*In vitro* characters are often used in combination with other agronomic traits for crop improvement programs. The availability of an efficient*in vitro* plant regeneration system is a prerequisite for the successful application of any biotechnological tool in plant breeding. Ikeuchi *et al*. (2013) provided an overview of callus and plant formation *in vitro* and described in detail the genetic and epigenetic mechanisms that induce or repress callus induction. An obvious challenge for tissue culture researchers is to develop*in vitro* technologies for faster, more predictable production of embryogenesis and plant regeneration. Despite all numerous technological advances, the successful application of plant tissue culture techniques to crop improvement is still dependent on understanding the genetic basis for '*in vitro* aptitude' that will help to predict the *in vitro*responses of genotypes for further selection. The influence of genotype on*in vitro* characters and the genetic basis of the *in vitro* response have been analyzed in several plant systems, including pearl millet (Pueschel *et al*., 2003; Satyavathi *et al*., 2006; Dodig *et al*., 2008; Chakravarthi *et al*., 2010; Etedal *et al*., 2012; Wang *et al*., 2013).

The study of the inheritance of genotypic differences is often complicated because of the occurrence of reciprocal differences (Powell and Caligari, 1987). The analysis of cytoplasmic factors in understanding the underlying modes of transmission of *in vitro* traits is more complicated than for nuclear factors. Various studies have examined the influence of cytoplasmic and/or maternal inheritance and the interaction between nuclear and cytoplasmic factors on *in vitro* somatic embryogenesis and morphogenesis in wheat (Ekiz and Konzak, 1991;Taylor and Veilleux, 1992; Bebeli, 1995; Ben-Amer and Boner, 1997), maize (Tomes and Smith, 1985; Willman *et al*., 1989; Ge *et al*., 1994), barley (Foroughi-Wehr *et al*., 1982; Powell and Caligari, 1987; Hou*et al*., 1994), rice (Abe and Futsuhara, 1991), red clover (Keyes *et al*., 1980), *Brassica* (Narasimhulu *et al*., 1989), tomato (Singsit and Veilleux, 1989),*Medicago* (Walton and Brown, 1988; Wan *et al*., 1988), *Allium ampeloprasum* L. (Silverstand *et al*., 1995) and*Desmodium* (Krottje *et al*., 1996).

The basis of the reciprocal differences in *in vitro* responses may be cytoplasmic factors, the maternal plant genotype or the segregation of nuclear factors. Discrimination between the first two phenomena becomes necessary in the analysis of reciprocal differences. When the female parent influences the phenotype of the progeny regardless of the latter's genotype, it is considered as maternal effect, whereas in cytoplasmic inheritance the mitochondrial DNAs transmitted to the progeny will have an effect on the phenotype of the organism (Henry *et al*., 1994). Powell (1988), using the biometrical analysis of Hayman (1954a), analyzed reciprocal differences in barley cultures attributed to component '*c*' (average reciprocal differences) to determine the percentage of green and albino plant production. This group also estimated component '*d*' (further reciprocal differences that were not accounted for by *c*) to assess the percentage corresponding to anthers and green plant production.

Among dicots, Narasimhulu *et al*. (1989) reported significant differences caused by cytoplasmic factors during shoot morphogenesis in *Brassica carinata*. Pueschel *et al*. (2003)analyzed the genetic control of the *in vitro* response of*Cyclamen persicum* genotypes with and without embryogenic potential. Based on the F_2_ segregation ratio, these authors postulated two major dominant genes with epistatic interaction for regeneration capacity.

Among monocots, Li *et al*. (2007) attempted to clarify the inheritance pattern of callus browning using reciprocal segregating F_2_ populations and two BC_1_populations derived from crosses between two inbred rice lines. Genetic analysis of callus browning and a map-based cloning strategy were used to locate the browning gene.

In wheat, Dodig *et al*. (2008)used correlation and path coefficient analyses to study associations between tissue culture and agronomic traits. Path coefficient analysis revealed that grain yield had the highest positive direct effect on regenerative calli and plant number per embryo. These authors observed a strong influence of genotype on tissue culture traits evaluated from immature embryos of wheat genotypes.

Mythili *et al*. (1997)suggested the presence of reciprocal differences for *in vitro*responses in pearl millet. However, no detailed analysis of such reciprocal differences has been reported. Subsequently, Satyavathi *et al*. (2006), in a study of the genetics of*in vitro* characters in pearl millet, reported the occurrence of reciprocal differences for all four of the *in vitro* characters they examined in reciprocal hybrids involving two inbred lines, P_3_(d_2_ dwarf) and P_4_ (purple pigmentation in plant parts) with phenotypic marker characters. This finding prompted use of the F_2_and backcross progenies of these particular crosses to establish the cause of the reciprocal differences based on the method by Hayman (1954a). The aim of the present study was to investigate the underlying genetic basis of reciprocal differences for callus characters and *in vitro* morphogenesis using biometrical genetical analyses in pearl millet.

## Material and Methods

### Plant material

Five inbred lines of pearl millet, *Pennisetum glaucum* (L.) R.Br., with different phenotypic marker characters were grown in the experimental farm of the Botany Department at Andhra University, Visakhapatnam, and have been maintained through selfing and sib mating for at least 8–10 generations. The five inbred lines were crossed in a diallel manner and the genetics of their *in vitro* response was studied (Satyavathi VV, 1998, PhD thesis, Andhra University, Visakhapatnam, India). Two parents, P3 (*d*_2_ dwarf measuring 80–120 cm) and P_4_(IP 3128 with purple pigmentation in the internodes, leaf sheath, midrib and margin) showing reciprocal differences for all of the *in vitro*characters studied, were crossed reciprocally and the F1 hybrids were back-crossed reciprocally with the respective parents. The F1s were selfed to produce eight F_2_ populations.

### Explant type and culture conditions

For callus initiation, segments of immature inflorescences at stage I (Mythili *et al*., 1997), still enclosed within the flag and boot leaves, were used. The stage of inflorescence was selected based on the relative distance between the flag and boot leaves and the two leaves subtending them. The nutrient medium and culture conditions used and the data collection for the *in vitro*characters were essentially as described by Satyavathi *et al*. (2006). Briefly, immature inflorescences were surface sterilized with 30% sodium hypochlorite solution for 30 min and washed three times with sterile distilled water. Each inflorescence measuring about 3–5 cm in length and having a pinhead-sized spikelet primordial was excised and cut into 0.5–0.6 cm discs. These discs (explants) were then placed on glass Petri dishes containing 20–25 mL of solidified Murashige and Skoog (1962) medium supplemented with 1 mg of 2,4-Dichlorophenoxyacetic acid (2,4-D) per ml at a density of 4–6 discs per dish. The dishes were sealed with parafilm and incubated at 26 ± 2º C in the dark for up to six weeks for callus induction. For regeneration, the callus pieces (embryogenic callus, referred to as E callus) were transferred to MSKB medium (MS medium with 0.25 mg/ml each of kinetin and 6-Benzyladenine) at the end of sixth week after inoculation. The cultures were incubated under 16 h:8 h light:dark photoperiod at a light intensity of 1600 lux provided by white florescent tubes.

### Experimental design

Six to ten plants from each of the two inbred lines, their two reciprocal F1s, about 100 plants from each of the four F1s and about 50 plants from each of the eight backcross populations were grown in the field in a randomized block design with two replications. Plants in the field as well as the arrangement of Petri dishes (each dish containing segments from one inflorescence representing a field grown plant) on the culture racks followed the same randomized pattern. The data were collected after six weeks of culture and included (1) the total quantity of calli, *i.e*., embryogenic and non-embryogenic calli measured using thin, transparent polythene graph paper and expressed in cm^2^, (2) the quantity of embryogenic calli expressed as a percentage of embryogenic callus area relative to the total callus area, (3) the callus growth rate defined as an increase in callus fresh weight at 3–6 week intervals, and (4) the frequency of regeneration scored as the number of plantlets produced per explant on MSKB medium after nine weeks.

### Data analysis

In a previous study (Satyavathi *et al*., 2006), we ran a 5x5 diallel analysis of five pearl millet inbred lines using four *in vitro* characters. The significance of the mean squares and the effects of general combining ability (GCA), specific combining ability (SCA) and reciprocals were analyzed using the combining ability analyses of method I described by Griffing (1956), based on Eisenhart's fixed effect model (model I) and the following assumptions: normal diploid segregation, absence of maternal effects, independent action of non-allelic genes, no multiple alleles, homozygous parents, independent distribution of genes between parents and inbreeding coefficient equal to 1.

In the present study, the data were further analyzed as described by Hayman (1954a) and the five main effects were calculated according to Mather and Jinks (1982). The components *c* (average reciprocal differences) and *d* (further reciprocal differences not accounted for by *c*) were estimated. The mean values for each of the four *in vitro* characters in the reciprocal hybrids (F_1_s) were analyzed using Student's *t*-test to check for individual differences in each of the ten hybrid combinations. Reciprocal differences between the crosses were examined using Student's*t*-test. The means and variances for individual plant data were estimated for each generation separately and a generation mean analysis was undertaken. The parents, F_1_, F_2_, BC_1_F_1_, BC_2_F_1_ and their reciprocals were used to fit a simple additive-dominance model in the generation means approach. A joint scaling test (Cavalli, 1952) was done. The model proposed by Hayman (1958), which estimates the mean (*m*), additive (*d*) and dominance (*h*) effects, and those caused by their interactions, *i*, *j* and*l*, was used. The genetic model for estimating additive- and dominance-genetic effects in the presence of maternal effects as given by Mather and Jinks (1982) was followed. This model provides estimates of the contribution of the cytoplasm (*c*), interaction between cytoplasm and the additive nuclear-determined effect (*cd*), interaction between cytoplasm and the dominance nuclear-determined effect (*ch*), maternal effects traceable to the additive genetic component (*dm*), and dominance effects of the maternal genotype (*hm*). The expectations for the family means in terms of genetic parameters for nuclear and cytoplasmic components, as well as for maternal effects as given by Powell and Caligari (1987), were applied to the four *in vitro* characters. The quantity of embryogenic calli and frequency of regeneration showed a good fit to the genetic model. The different parameters were estimated from the means of the 16 generations and a comparison of the generation mean with the expected values was done using the joint sealing test. The significance of each of the estimated parameters and the adequacy of the additive-dominance model were tested using standard procedures (Mather and Jinks, 1982).

## Results

**Figure 1:**
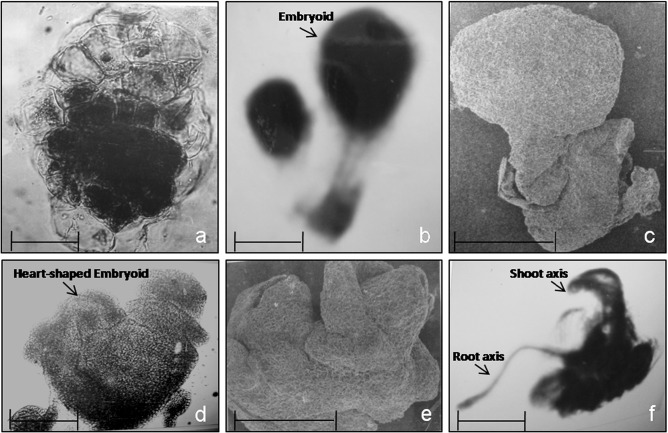
Various developmental stages during somatic embryogenesis in pearl millet. (A) A single ovoid proembryoid, (B) Stalked globular embryoids, (C) Scanning electron micrograph of a stalked globular embryoid, (D) Heart-shaped somatic embryo, (E) Scanning electron micrograph of a somatic embryo with collar-like scutellum (200x), and (F) Somatic embryo with root and shoot primordia. Scale bar =10 μm.

In the present study, *in vitro* regeneration of pearl millet was obtained by culturing immature inflorescences as described earlier (Mythili *et al*., 1997; Satyavathi *et al*., 2006). Plant regeneration was via somatic embryogenesis. Histological examination of the explants showed cellular proliferation beneath the outer layer of rachis and also from the base of the spikelet primordia. The calli obtained were of two types: embryogenic (E callus) and non-embryogenic (NE callus). At the end of the sixth week after inoculation, segments of embryogenic calli were transferred to regeneration medium. Embryoid formation was observed one week after the transfer of embryogenic calli to regeneration medium. These embryoids first appeared as ovoid masses of cells that later developed into globular proembryoids to subsequently form heart-shaped embryoids. At this stage, the somatic embryoids appeared as white opaque protuberances with collar-like structures; these somatic embryos showed root-shoot differentiation and developed into plantlets (Figure 1).

**Figure 2:**
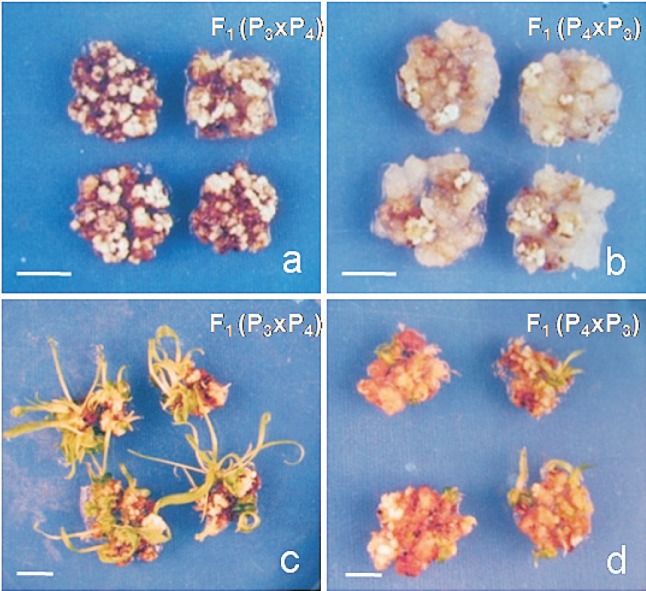
Differences in the quantities of embryogenic (A) and non-embryogenic (B) portions of callus and in the number of regenerated plantlets in the corresponding hybrid F_1_(P_3_xP_4_) (C) and reciprocal hybrid F_1_(P_4_xP_3_) (D) involving the P3 and P4 inbred lines. Scale bar =100 μm.

The calli obtained from P_3_ and P_4_ parents were distinguishable, with the P_3_ parent showing highly compact, nodular calli while the P_4_ parent showed mostly crystalline, transluscent calli. In the hybrids involving these two parents, the reciprocal differences were significant for all of the four *in vitro* characters studied. The quantity of embryogenic and non-embryogenic portions of calli and the number of regenerated plantlets in the reciprocal hybrids involving P_3_ and P_4_ are shown in Figure 2.

The Hayman (1954a) analysis for a 5x5 diallel set of pearl millet lines revealed items '*c*' and '*d*' to be significant for the quantity and frequency of regeneration of embryogenic calli, whereas only item '*d*' was significant for total quantity of calli and growth rate (Table 1). Among the ten hybrid combinations from a 5x5 diallel set, the reciprocal differences were significant for all of the four *in vitro* characters in the cross involving P_3_ and P_4_inbreds. In the remaining hybrids, the reciprocal differences were significant for only one or two characters (Table 2). Consequently, the cross involving P_3_ and P_4_ was chosen for a detailed analysis of the reciprocal differences. One set of crosses, viz., P_3_xP_4_ and its reciprocal P_4_xP_3_, was used to produce the reciprocal F_2_s as well as reciprocal backcrosses, thus providing 16 families. The average values for each of the four *in vitro* characters of the 16 families were examined.

**Table 1:** Hayman analyses of variance for the four *in vitro*characters obtained from a 5x5 diallel analysis.

		Mean squares			
Item	Degrees of freedom	Total callus quantity	Embryogenic callus quantity	Callus growth rate	Regeneration frequency
*a*	4	0.0209*	155.5555*	0.0394*	6.6107*
*b*	10	0.0078	61.6698*	0.0151*	4.6833*
*b1*	1	0.0006	1.5819	0.0505	3.0489*
*b_2_*	4	0.0185*	61.4082*	0.0214*	5.6812*
*b_3_*	5	0.0074	73.8966*	0.0029	4.2119*
*c*	4	0.0134	78.6379*	0.0023	0.4479*
*d*	6	0.0057*	13.0646*	0.0075*	0.7657*

*a* – additive genetic variation, *b* – dominance variation, *b_1_* – mean dominance deviation, *i.e*., the overall difference between the F_1_s compared to their mid-parent values,*b_2_* – the variation in mean dominance deviations of the F_1_s from their mid-parent values within each array, over arrays, *b_3_* – dominance deviations that are unique to individual F_1_s,*c* – average reciprocal differences,*d* – further reciprocal differences not accounted for by *c*. Each item was tested against its own block interaction.

* p < 0.05.

**Table 2:** Reciprocal differences for the four *in vitro* characters in the different crosses (F_1_s).

	Total callus quantity		Embryogenic callus quantity		Callus growth rate		Regeneration frequency	
Cross	t value	df	t value	df	t value	df	t value	df
P_1_xP_2_ vs P_2_xP_1_	1.1157	49.5	2.0040*	55.9	0.8185	10	0.2199	55
P_1_xP_3_ vs P_3_xP_1_	3.8631*	66.9	2.9768*	76.0	0.0207	11	0.8213	61
P_1_xP_4_ vs P_4_xP_1_	1.1050	48.0	1.7026	72.0	1.6554	9	2.0053*	61
P_1_xP_5_ vs P_5_xP_1_	1.6678	78.5	0.9592	82.0	3.9175*	9	0.9362	56
P_2_xP_3_ vs P_3_xP_2_	2.4558*	90.0	0.1107	71.3	1.0071	10	0.8820	66
P_2_xP_4_ vs P_4_xP_2_	0.1946	56.7	0.4315	79.5	1.9092	10	0.3663	49
P_2_xP_5_ vs P_5_xP_2_	2.0833*	70.0	0.2652	66.0	0.8549	6.2	0.8589	67
P_3_xP_4_ vs P_4_xP_3_	3.7678*	68.7	4.0852*	79.0	3.7583*	9	2.0285*	49
P_3_xP_5_ vs P_5_xP_3_	6.2262*	68.7	3.0623*	63.5	1.5066	10	0.1634	48
P_4_xP_5_ vs P_5_xP_4_	3.7778*	58.0	0.6842	50.6	0.8956	10	0.5583	51

df calculated from Behren-Fisher's conversion.

* p < 0.05.

Students *t*-test, used to detect differences in the means of each of the four *in vitro* characters, revealed significant differences between P_3_ and P_4_, between reciprocal F_1_s (P_3_xP_4_ and P_4_xP_3_), and between reciprocal backcrosses for BC_1_ (callus growth rate) and BC_2_(total callus quantity) (Table 3). A two-way ANOVA used to assess the differences for each character revealed significant differences (p < 0.05) among the 16 families as expected, but no differences between the replications (p > 0.05); a significant interaction factor (p < 0.01) was also observed (data not shown).

**Table 3:** Comparison of the average values of the two parents and corresponding reciprocal populations of F_1_s, F_2_s and backcrosses.

	Total callus quantity		Embryogenic callus quantity		Callus growth rate		Regeneration frequency	
Population	t value	df	t value	df	t value	df	t value	df
Parents P_3_ vs P_4_	7.1459*	10	9.4947*	10	9.9550*	10	8.6273*	10
F_1_(P_3_xP_4_) vs F_1_ (P_4_xP_3_)	3.1727*	9	2.4650*	6.7	3.2509*	5.2	2.9635*	9
F_2_(P_3_xP_4_) x (P_3_xP_4_) vs (P_4_xP_3_) x (P_4_xP_3_)	1.1015	198	1.8707	198	1.2970	192	1.0463	197
F_2_(P_3_xP_4_) x (P_4_xP_3_) vs (P_4_xP_3_) x (P_3_xP_4_)	0.7636	187	0.9143	185	1.0214	146.3	0.5181	173
BC_1_(P_3_x(P_3_xP_4_) vs (P_3_xP_4_)xP_3_)	0.1773	83	0.3511	83	2.7090*	50.2	0.5573	81
BC_1_(P_3_xP_4_xP_3_) vs (P_4_xP_3_)xP_3_)	0.3147	96	0.6032	96	3.4493*	52.1	1.9679	93
BC_2_P_4_x(P_3_xP_4_) vs (P_3_xP_4_)xP_4_	2.5505*	72.8	1.3323	75	1.8129	64.7	0.5853	74
BC_2_P_4_x(P_4_xP_3_) vs (P_4_xP_3_)xP_4_	2.6741*	63.6	2.7113*	63.4	0.0010	65	1.5185	65

df calculated from Behren-Fisher's conversion.

* p < 0.05.

Embryogenic callus quantity and regeneration frequency showed a good fit to the genetic model, although the chi-square test of the goodness of fit was not significant (p > 0.01); the other two characters did not fit the model (p < 0.01) (Table 4). For embryogenic callus quantity, the parameters *d*, *j* and*cd*, representing the additive effect, additive x dominance interactions and the interaction between cytoplasmic and nuclear-determined effects, respectively, were significant. For the regeneration frequency, all of the parameters that provided estimates of the main effects and that represented the nuclear genetic contributions to the family means were found to be significant. The dominance increment of loci (*h*) for the nuclear genetic contribution was positive, while the '*l*' increment for pairs of loci was negative. The maternal effects included an additive genetic component (*dm*) and dominance (*hm*); the former affected total callus quantity, whereas the latter affected total callus quantity, embryogenic callus quantity and regeneration frequency.

**Table 4:** Estimates of the genetic parameters for nuclear and cytoplasmic components and chi-square values for the four in vitro characters

	Total callus quantity		Embryogenic callus quantity		Callus growth rate		Regeneration frequency	
Parameter	Estimate	t value	Estimate	t value	Estimate	t value	Estimates	t value
*m*	1.4784 ±0.2777	5.3227	15.6168 ± 4.7491	3.2884*	1.1903 ±0.1914	6.2183	0.1903 ± 0.3327	0.5720
*d*	0.7655 ±0.2403	3.1847	21.0579 ± 4.5716	4.6063*	−0.5212 ± 0.1520	−3.4281	−0.7862 ± 0.2229	−3.5268*
*h*	1.7179 ±0.4257	4.0336	9.3529 ± 7.5090	1.2455	−0.8066 ± 0.3155	−2.5565	1.9158 ± 0.5298	3.6163*
*i*	0.5156 ±0.2522	2.0447	4.0013 ± 4.1421	0.9660	0.0381 ±0.1981	0.2024	1.0831 ± 0.3054	3.5467*
*j*	−1.0542 ± 0.4326	−2.4370	−24.0144 ± 8.4691	−2.8355*	0.5554 ±0.2704	2.0539	1.5378 ± 0.3229	4.7611*
*L*	−0.7715 ± 0.2064	−3.7374	−3.1369 ±4.1760	−0.7511	1.0253 ±0.1751	5.8563	−0.7404 ± 0.2583	−2.8666*
*c*	0.3394 ±0.1711	1.9837	6.9015 ± 3.6373	1.8974	−0.0755 ± 0.1276	−0.5918	−0.2864 ± 0.1416	−2.0229
*cd*	0.2090 ±0.1045	1.9989	−4.1908 ± 1.7173	−2.4403*	0.2051 ±0.0743	2.7596	0.0032 ± 0.1539	0.0206
*ch*	−0.6605 ± 0.3354	−1.9694	−14.4373 ± 7.2165	−2.0006	0.2627 ± 0.2499	1.0510	0.5399 ± 0.2716	1.9879
*dm*	0.1751 ±0.0937	1.8689	−1.2915 ± 1.4846	−0.8699	−0.0779 ± 0.0535	−1.4556	−0.0626 ± 0.1029	−0.6077
*hm*	0.1218 ±0.0987	1.2340	1.3071 ± 1.6663	0.7844	−0.1851 ± 0.0571	−3.2379	0.1472 ± 0.1235	1.1917
χ^2^(5)	24.8797		13.9496		35.2062		14.8993	

*cd* – interaction between cytoplasm and the additive (d) nuclear-determined effect, *ch* – interaction between cytoplasm and the dominance (h) nuclear-determined effect,*d* – additive genetic component, *dm*– maternal effects traceable to the additive genetic component,*h* – dominance, *hm* – dominance effects of the maternal genotype, *i* – additive by additive interaction, *j* – additive by dominance interaction, *l* – dominance by dominance interaction,*m* – population mean. Chi-square test for the goodness of fit was not significant (p > 0.01).

* p < 0.01.

## Discussion

Cytoplasmic inheritance results in persistent reciprocal differences and reflects an unequal contribution of cytoplasmic determinants from male and female gametes to the zygote (Jinks, 1964). The maternal tissue effects are transient and change with the genotype of the mother. Since the cytoplasmic component includes either the chloroplast or mitochondrial DNA, a distinction between maternal and cytoplasmic modes of inheritance becomes necessary. This distinction is also important in order to manipulate the particular character, depending on its inheritance patterns. An in-depth study of the progenies other than the parents and F_1_s (that is F_2_s and backcrosses) is required when fitting genetic models that allow estimation of cytoplasmic and maternal effects and their adequacy in describing the observed reciprocal differences.

In a previous study, the ANOVA of genetic components from a 5x5 diallel cross revealed that GCA and SCA were significant (p < 0.05) for the four *in vitro* characters studied, namely total callus quantity, embryogenic callus quantity, callus growth rate and frequency of regeneration (Satyavathi VV, 1998, PhD thesis, Andhra University, Visakhapatnam, India). GCA analysis indicated P_4_ as the best general combiner for total callus quantity and growth rate, P_3_ for embryogenic callus quantity, and P_1_ as well as P_3_ for regeneration frequency. SCA analysis revealed that some of the crosses (P_1_xP_3_, P_3_xP_4_ and P_3_xP_5_) were the best specific combinations for one or more of the *in vitro* characters studied. In general, hybrids involving two poor general combiners showed better SCA effects. For callus growth rate and regeneration frequency, combinations of one better and one poor combiner produced better SCA effects. Similar GCA and SCA effects have been reported in rice (Abe and Futsuhara, 1991), maize (Petolino and Thompson, 1987), winter wheat (Ou *et al*., 1989),*Allium ampeloprasum* (Silverstand *et al*., 1995), *Helianthus annuus* L (Sarrafi *et al*., 1996), eggplant (Chakravarthi *et al*., 2010) and rapeseed (Etedal *et al*., 2012).

Based on Hayman's analysis, significant additive genetic variation was observed for all of the four *in vitro* characters. The most striking feature observed was the presence of highly significant average reciprocal effects (*c*) for embryogenic callus quantity and regeneration frequency. The *d* component was also significant for all of the characters studied.

Our results revealed that embryogenic callus quantity and regeneration frequency showed a good fit to the genetic model of Mather and Jinks (1982), whereas for the other two characters (total callus quantity and growth rate), this model was not a good fit, as indicated by the highly significant *X* ^2^ values (Table 4).

For embryogenic callus quantity, the estimates (*additive*) and (*additive* x *dominance*) were significant among the nuclear parameters. The interaction between the cytoplasm and nuclear additive component ('*cd*') was also significant. These findings suggested that the reciprocal differences for embryogenic callus quantity could be attributed to the interaction between cytoplasmic and nuclear determinants. Narasimhulu *et al*. (1989)reported a similar observation for shoot morphogenesis in *Brassica*spp. and suggested that both cytoplasm and nuclear genomes contain determinants important for temporal regulation and co-ordinated synthesis of factors that promote shoot morphogenesis. Janila *et al*. (2013), based on genetic analyses of resistance to late leaf spot (LLS) in interspecific hybrids of groundnut, reported that a combination of nuclear and maternal gene effects was involved in the resistance factor.

For regeneration frequency in which the additive-dominance model was adequate, five out of the 11 estimates were significant, confirming the predominant role of nuclear gene control in the expression of this character. The positive (*h*) increment of loci and the negative (*l*) increments for pairs of loci suggested the occurrence of non-allelic interaction of the duplicate kind in the expression of this character. This type of duplicate epistasis was considered to be associated with characters expressed in directional selection (Powell and Caligari, 1987). As inferred from the work of Satyavathi *et al*. (2006), regeneration frequency is associated with the *d_2_* gene that controls the dwarf nature of the plant. The suggestion by Powell and Caligari (1987) regarding the operation of directional selection is consistent with the origin of P_3_(*d_2_* dwarf line) developed by Burton and Fortson (1966) and the association or linkage between the *d_2_* locus and loci controlling*in vitro* regeneration frequency. The P_3_ line was the result of breeders selecting for homozygosis dwarf stature (*d_2_d_2_*), *i.e*., unidirectional selection for reduction in height. Since at least some of the loci controlling *in vitro* regeneration are linked to the*d_2_* locus, the selection for dwarfism might also have indirectly affected the linked loci for regeneration.

Both linkage and selection (direct and indirect) for the two characters (dwarf nature and regeneration) might have resulted in the characteristic and visually identifiable callus morphology noted in the dwarf parent. For *Cyclamen persicum*, Pueschel *et al*. (2003) postulated a hypothesis of two dominant epistatic genes controlling the capacity for regeneration and stated that the trait can easily be integrated into other genotypes of economic interest by crossings. Moreover, linked DNA-markers would enable early selection of desired genotypes capable of regenerating somatic embryos and would spare the time and labor involved in*in vitro* screening. Dodig*et al*. (2008) studied the correlation between tissue culture and agronomic traits in wheat. Agronomic traits with highly positive direct effects on tissue culture traits were considered as suitable predictors of good*in vitro* plant regeneration. These authors found productive tillering to have a significant (positive) direct effect on all tissue culture traits.

Our study revealed that for two characters, embryogenic callus quantity and regeneration frequency, the former showed a greater proportion of cytoplasmic nuclear interaction whereas the latter showed a greater role of nuclear factors.Mathias *et al*. (1986)reported the role of specific interactions between the nuclear and cytoplasm in determining tissue culture responses in Chinese spring wheat. The possibility that regeneration from tissue culture is partly controlled by specific nuclear-cytoplasmic interactions has also been suggested by Henry *et al*. (1994).

Since the regeneration frequency is dependent upon embryogenic callus quantity, the*d_2_* cytoplasm might also contribute (in addition to directional selection and linkage) to the characteristic response and morphological appearance of the callus in dwarf parents. This conclusion raises the possibility of manipulating these *in vitro* characters through the selection of appropriate lineages, despite their complex inheritance patterns.
